# Lessons From Astronomy and Biology for the Mind—Copernican Revolution in Neuroscience

**DOI:** 10.3389/fnhum.2019.00319

**Published:** 2019-09-19

**Authors:** Georg Northoff

**Affiliations:** Cellular and Molecular Medicine Faculty of Medicine, University of Ottawa Institute of Mental Health Research, Ottawa, ON, Canada

**Keywords:** physics, biology, neuroscience, common currency, copernican turn

## Abstract

Neuroscience made major progress in unravelling the neural basis of mental features like self, consciousness, affect, etc. However, we nevertheless lack what recently has been described as “missing ingredient” or “common currency” in the relationship between neuronal and mental activity. Rather than putting forward yet another theory of the neural basis of mental features, I here suggest a change in our methodological strategy how to approach the brain, that is, our view or vantage point of the brain. Learning from astronomy (Copernicus) and biology (Darwin), I suggest that we may want to change our currently pre-Copernican vantage point from within brain to a post-Copernican vantage point from beyond brain. Such post-Copernican vantage point from beyond brain allows us taking into view that what happens beyond the brain itself, e.g., the world, and how that shapes the brain and its neural activity, e.g., world-brain relation. We then lend empirical support to the world-brain relation by converging it with Karl Friston’s free energy principle that, as we see it, provides a neuro-ecological and therefore post-Copernican view of the brain. That, in turn, allows us taking into view that mental features are shaped by both world and brain and are therefore truly neuro-ecological rather than merely neuronal. This raises the question for the link, e.g., the “missing ingredient” or “common currency” of world brain relation and mental features. Recent empirical evidence suggests that temporo-spatial dynamics may provide such link as it characterizes both the world-brain relation’s free energy and mental features, e.g., their spatiotemporality as described in philosophy. Taken together, I here advocate a change in our methodological strategy on how to approach the brain, that is, a shift from a pre-Copernican vantage point from within brain to a post-Copernican vantage point from beyond brain. The latter allows us taking into view that what happens beyond the brain in the world and how that shapes the brain in such a way that it can yield mental features. This amounts to nothing less than a Copernican turn or revolution in neuroscience akin to the ones in astronomy (Copernicus) and biology (Darwin).

## Introduction

### Mental Features—How Can We Reconcile World and Brain?

The mind and its various mental features present us with a puzzle. On the one hand, mental features like self, consciousness, and affect (and others) can be characterized by an experience or perception of specific events or objects and even the own self as part of the wider world beyond ourselves, e.g., body and brain. Taken in such sense, mental features exhibit a strong ecological component. On the other hand, recent research in neuroscience clearly demonstrates a neural basis of the various mental features in the brain. One would consequently assume that mental features are neuronal rather than ecological.

How can we reconcile both mental, e.g., ecological and neuronal views of mental features in our empirical research in neuroscience (while refraining from any ontological metaphysical assumptions; see below)? One way to do so is to reduce mental features to the neuronal mechanisms of the brain. This is reflected in various excellent neuroscientific theories of mental features like consciousness and self. Among others, these include the Integrated Information Theory (IIT; Tononi et al., 2016), the Global Neuronal Workspace Theory (GNWT; Dehaene et al., 2014, 2017), and the Temporo-spatial Theory of consciousness (TTC; Northoff, 2013, 2014a,b, 2016a,b,c,d, 2018; Northoff and Huang, 2017).

The same also holds for other mental features like self where neuronal accounts of cognitive (Churchland, 2002), dynamic pattern (Gallagher, 2005), affective (Panksepp, 1998a,b), attentional (Sui and Humphreys, 2015), embodied (Gallagher, 2005; Thompson, 2007; Hu et al., 2016), and temporo-spatial (Northoff, 2016a,b,c, d, 2017) theories of self have been suggested. Finally, affect has also been the focus where, rather than reducing it, neuronal and mental features are conceived as two sides of one and the same activity (which, metaphysically, presupposes dual-aspect monism)—this has recently been suggested by Mark Solms who conceives affect as most basic and primary manifestation of consciousness and mental features (Solms, 2017, 2018, 2019; see also Damasio, 2018).

Yet another methodological strategy on how to reconcile ecological and neuronal views of mental features is to change our approach to the brain. Specifically, one may want to take into view that what happens beyond the brain itself, e.g., in world and body, and how that shapes the brain itself in such way that it can yield mental features. Such neuro-ecological (rather than purely neuronal) view of the brain, in turn, may allow us to account for both ecological and neuronal aspects of mental features. Even more important, we can then take into view that what has been recently described as “missing ingredient” (Lamme, 2018) or “common currency” (Northoff, 2019) of neuronal and mental features. The main goal in the present article consists in sketching such alternative methodological strategy in our approach to the brain and how it yields mental features.

### Main and Specific Aims—Copernican Revolution in Neuroscience

My main aim is to demonstrate that neuroscience can learn from both astronomy and biology in their Copernican turns. Copernicus changed our view of earth (see below) which allowed him to take into view a novel and different relation of universe and earth. Analogously so in the case of Darwin. He changed our view of human species which enabled him to take into view our relation to evolution (see below for details on both Copernicus and Darwin). I now suggest the same kind of Copernican turn or revolution with regard to the brain. We may want to change our currently pre-Copernican view of the brain and replace it by a post-Copernican view. That, as I suggest, will enable us to take a novel post-Copernican view how the brain is related to the world, e.g., world-brain relation (see below for details; Northoff, 2016a,b,c,d, 2018). Importantly, this, in turn, makes possible to reconcile ecological and neuronal view of mental features as truly neuro-ecological rather than as merely neuronal.

My suggestion amounts to nothing less than the claim for a Copernican revolution in neuroscience (analogous to the ones in astronomy and biology). Note that I conceive such Copernican revolution in merely empirical terms of neuroscience. Hence, I only focus on the methodological strategy, e.g., our view or vantage point (see below) with regard to the brain in purely empirical terms, that is, how we can approach and understand the brain’s neuronal features as we observe and investigate them in neuroscience. In contrast, I refrain from more philosophical claims of an epistemological (as, for instance, Kant suggests; Kant, 1781/1998), metaphysical, or ontological (Whitehead, 1929/1978; Sherburne, 1983; Northoff, 2016a,b,c,d). Copernican revolution (see also Northoff, 2018, for discussing the Copernican revolution in more detail in the context of the mind-body problem or world-brain problem).

Refraining from such wider senses of the Copernican revolution beyond the merely empirical territory of neuroscience entails that I here do not address any kind of philosophical problems like the “explanatory gap” (Levine, 1983), “hard problem” (Chalmers, 1996), or mind-body problem (see also Northoff, 2018 for the dissolution of the mind-body problem and its replacement by the world-brain problem). Accordingly, my understanding of the Copernican revolution in this article is purely empirical and limited to neuroscience (rather than psychology as Sigmund Freud has also been attributed a Copernican revolution with respect to the relation of consciousness and unconsciousness; see Weinert, 2013) which, as I see it, is akin to the revolutions in both astronomy (Copernicus) and biology (Darwin).

The first specific aim consists in briefly describing the basic features of the Copernican revolutions in both astronomy (Copernicus) and biology (Darwin). That will provide the basis for the second specific aim, that is, the comparison of pre- and post-Copernican views of the brain. The third specific aim is to sketch a post-Copernican view of the brain by suggesting a neuro-ecological view of the brain in terms of Karl Friston’s free energy principle (Friston and Stephan, 2007; Friston, 2010; Bruineberg et al., 2018a,b). That sets the basis for the fourth specific aim that consists in outlining (albeit tentatively) a post-Copernican view of mental features as neuro-ecological and temporo-spatial.

## Part I: Pre- vs. Post-Copernican Vantage Points in Astronomy and Biology

### Vantage Point—Egocentric vs. Allocentric

What is a vantage point? I here consider the concept of vantage point in its original definition as a “position or stand point from which something is viewed or considered” (*Oxford Dictionary*). Taken in this sense, the concept of vantage point comes close to those of point of view or viewpoint. The chosen vantage point may provide a specific view or viewpoint that includes a wide range of phenomena while excluding others.

Let us take the example of viewing a city. One walks around within a city. That allow us to see the details of, for instance, the mosaic on the door of the big gothic cathedral. In contrast, we remain unable to take into view the cathedral as such and how it is integrated and thus fits into its respective context, i.e., the city as whole. Specifically, the relationship between city as whole and the cathedral as part of it remains opaque to us. The cathedral looks very special to us in such viewpoint since it is not related at all to the rest of the city and its spatiotemporal coordinates—our view thus highlights the specialness of the cathedral and its dichotomy with the city. Such vantage point from within city takes the city itself including the cathedral as center thus entailing what can be described as “vantage point from within city (and cathedral).” Such viewpoint corresponds well to the egocentric “vantage points from within earth and humans” in astronomy and biology prior to Copernicus and Darwin (see below).

That changes, once one moves to the mountain nearby from which one can view the city as a whole including the big cathedral. Now, we can take into view how the big cathedral is part of the overall spatiotemporal coordinates of the city that is, how well it integrates and aligns to its respective context and the city as a whole. The spatiotemporal scope and range of our view is thus extended beyond the cathedral—this is possible by taking a what we describe as “vantage point from beyond city (and cathedral).” As we will see below, that corresponds well to the allocentric vantage points Copernicus and Darwin introduced, the “vantage points from beyond earth and humans.”

### Pre- vs. Post-Copernican Vantage Points in Astronomy—Earth and Universe

**Figure 1:**
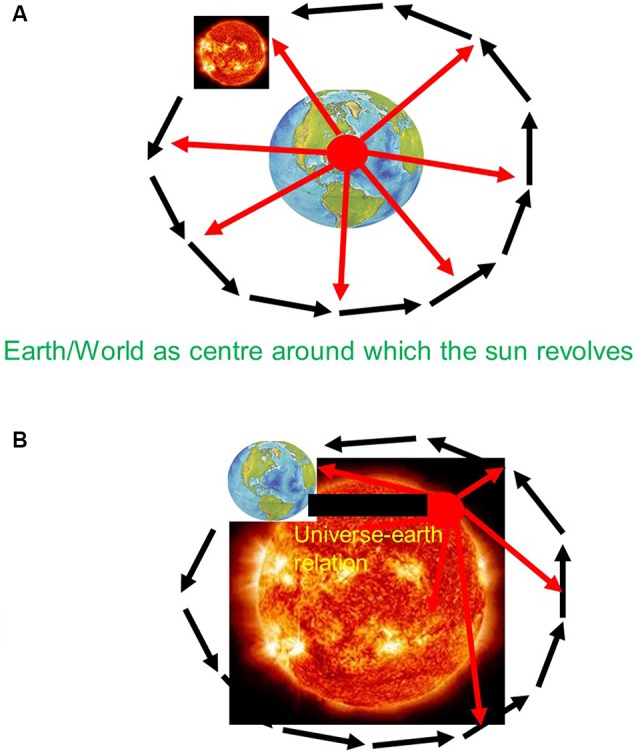
**(A)** Pre-Copernican—Geo-centric view with Vantage point from within earth. **(B)** Post-Copernican—Helio-centric view with Vantage point from beyond earth.

Before Copernicus, the universe did include earth and other planets. However, the earth was not just another planet besides the others within the universe as a whole. Instead, earth was conceived special when compared to the other planets and the rest of the universe. Specifically, the earth was supposed to hold together the universe when being attributed the role of the center around which all other planets move in the periphery. There was thus a clear center-periphery dichotomy between earth and non-earth—the earth was deemed special entailing a geocentric and consequently egocentric view of the universe, a “vantage point from within earth” (see also Northoff, 2018; see Figure 1A).

The specialness of earth was further supported by its non-moving character. Being conceived as center that holds the whole universe together, the earth itself was not supposed to change as any such change would lead to the breakdown of the universe. The earth was thus conceived in an a-temporal way—the earth was assumed to be eternally present without any change in space and time. Together, the earth was characterized by specialness, e.g., its role as center, and its dichotomy to the universe, e.g., a-spatiotemporal vs. spatiotemporal.

The view of the universe changed with Copernicus which was empirically confirmed by his successors Kepler, Bruno, Gallilei, and Newton (Weinert, 2013; Northoff, 2018). Copernicus introduced a different view of earth within the universe. He attributed the observed movements to earth itself. Rather than sun and all other planets revolving around earth as center of the universe, he suggested the opposite namely that the earth revolves around the sun as center of universe. This led to the replacement of the geocentric and egocentric view of the universe by a heliocentric and allocentric view of the relationship of earth and universe.

In the case of a heliocentric framework, the earth loses its specialness in terms of its position, its role, and its temporal features. The earth is no longer the center of the universe around which all other planets revolve. Instead, the earth is replaced by the sun—the special position of earth is thus lost. Moreover, it is no longer the earth that holds together the universe but the sun—the central role of earth for the universe is also lost. Finally, the a-spatiotemporal nature of earth as not being subject to change is replaced by attributing movements to earth as it revolves around the sun—the earth is thus characterized by the same spatiotemporal features as the rest of the universe albeit in different degrees, e.g., in a smaller scale. Together, the specialness and dichotomy of earth in the pre-Copernican view are replaced by non-specialness of earth and intrinsic relationship of earth and universe.

How was it possible for Copernicus to take into view the non-specialness of earth and its intrinsic relationship with the universe? He abandoned the traditional vantage point from within earth and replaced it by one that allowed him to take into view that what happens beyond the earth itself within the universe and how that shapes the earth itself, e.g., its movements within the universe. Like the tourist walking to the nearby hill to view city and cathedral, Copernicus shifted the vantage point from within earth to what I describe as “vantage point from beyond earth” (see Figure 1B).

### Pre- vs. Post-Copernican Vantage Points in Biology—Human and Non-human Species

How about biology? Darwin is often credited with bringing about a Copernican turn which lead to a scientific (and metaphysical) revolution in biology (Ruse, 2009; Weinert, 2013). Before Darwin, humans were considered special when compared to non-human species. Humans were regarded the center of the world with capacities vastly superior to the ones of non-human species. This led to the assumption of a special role of humans as only they were attributed soul and mind which enabled them to be in special contact with God as creator of the world. Since God does not change and is therefore a-spatiotemporal, humans and, more specifically, their soul or mind must also be a-spatiotemporal (as otherwise they could not be in contact with God) this entailed dichotomy of mind and world.

Both specialness of humans and their dichotomy to the rest of the world can be taken into view only when presupposing a “vantage point from within humans.” That all changed with Darwin though. He presented empirical evidence that humans are part of the same evolution as non-human species. The specialness of humans was thus lost and replaced by their non-specialness.

Even more important, Darwin showed that both humans and non-humans are subject to the same principles in the world, e.g., natural selection, throughout space and time in their evolution. This allowed Darwin to take into view the intrinsic relationship of humans and non-humans including their commonly shared spatiotemporal features. Accordingly, Darwin thus able to take into view that happens beyond human species in the world including non-human species and how that shapes and relates to the humans themselves.

Look beyond humans themselves which presupposes a “vantage point from beyond human.”

## Part II: Pre- vs. Post-Copernican Vantage Point in Neuroscience

### Vantage Point From Within Mind—From Philosophy to Psychology and Cognitive Science

The famous 17th century philosopher Descartes is considered the main source of the dualism of mind and body. The body as part of the wider world can be observed in time and space, that is, at discrete points in time and space, and operates in a purely mechanical way like a machine. That, in contrast, is not the case in consciousness and other mental features like self. The mental features are neither spatiotemporal, e.g., they a-spatiotemporal as traditionally conceived in philosophy. Nor are mental features mechanical like body and world. We, therefore, cannot attribute mental features to the body as part of the world but to the mind.

Together, the mind is special as it alone can mediate mental features like consciousness, affect/emotional feelings, and self—this reflects the specialness of mind. At the same time, the mind can be characterized by its dichotomy to body/world with seemingly no relationship between both. For that reason, Descartes conceived mind and body/world as separate existences and realities—this led him to the famous mind-body dualism which, at the same time, implies dualism of mind and world (McDowell, 1994; Northoff, 2018).

**Figure 2:**
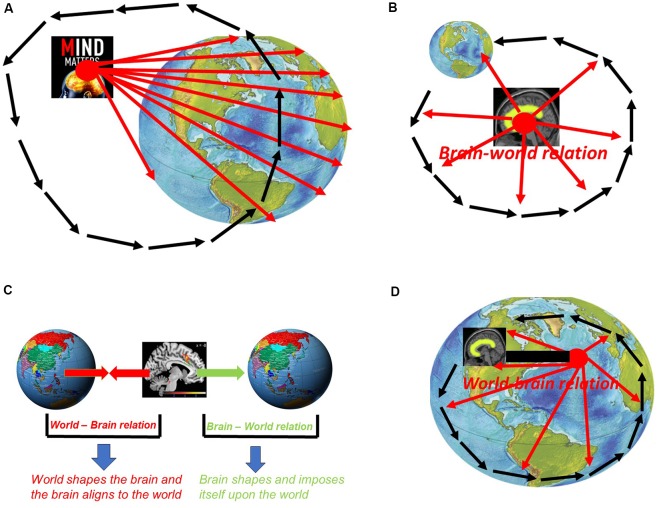
**(A)** Vantage point from within mind: mento-centric and pre-Copernican stance. **(B)** Vantage point from with brain: neuro-centric and ego-centric view with brain as center of the world. **(C)** World-brain relation vs. brain-world relation. **(D)** Vantage point from beyond brain: allo-centric view of the brain—post-Copernican.

How is it possible to conceive the possibility of mind-body dualism including specialness of mind and its dichotomy to body and world? This presupposes a vantage point that takes the mind itself as its center or “primary location” in the same way pre-Copernican astronomists and biologists took earth and humans as their viewpoints. The assumption of the specialness of mind and its dichotomy to body/world thus presupposes a “vantage point from within mind” (Northoff, 2018). Taking the mind as reference for mental features, such vantage point from within mind leads to a mento-centric and ego-centric view of mental features as being special and dichotomous to body and world (see Figure 2A).

One may now want to argue that such mind-body dualism is just a merely philosophical problem. Current research in psychology and cognitive science goes beyond that by showing how the mind operates, displays different functions like consciousness, cognition, self, and affect/emotion, and uses certain computational principles and mechanisms. That does not change the basic methodological presupposition though, that is, the egocentric vantage point from within mind. True psychology and cognitive science shift from the metaphysical domain of philosophy to the empirical domain. That by itself does not change the methodological strategy how to approach the mind though.

Even an empirical approach to the mind can still take the mind itself as center and conceive the latter as special. That is, for instance, the case when one attributes specific psychological processes or computational mechanisms to mental features which stand in dichotomous relation to those of non-mental features in body and world. The mento- and thus egocentric character of the methodological approach to the mind is thus more or less preserved in psychology and cognitive science—they thus presuppose a vantage point from within the mind.

### Vantage Point From Within Brain—Neuroscience

One may now be tempted to say that we know better these days. The assumption of mind has been disputed in both philosophy and even more so in neuroscience in our time. There is no mind anymore, mental features like consciousness and self are based on the brain and are thus physical or better neuronal rather than primarily mental, e.g., non-physical. Mind-body dualism is thus replaced by monism with either materialism/physicalism, panpsychism (Tononi and Koch, 2015), or dual-aspect monism (Solms, 2017, 2018, 2019).

The recognition of the neural basis of mental features has led to a search for their neuronal mechanisms. In the debate about, for instance, consciousness, this has led to the search for the neural correlates of consciousness (the NCC; Chalmers, 2001; Crick and Koch, 2003; Koch, 2004; Aru et al., 2012; de Graaf et al., 2012; Northoff, 2013, 2014a,b; Solms, 2018, 2019). The NCC has been defined as the minimum neuronal mechanisms jointly sufficient for any one specific conscious percept (Crick and Koch, 1990; Koch, 2004). Recent progress in consciousness research further introduces two refined interpretations of the NCC as: (1) the content-specific NCC, which determines a particular phenomenal distinction with an experience; and (2) the full NCC, which supports conscious experiences in their entirety, irrespective of the contents (Koch et al., 2016).

The NCC assumes special neuronal features within the brain itself. These special yet not fully clear neuronal features (see below) are supposed to underlie consciousness; this distinguishes them from other neuronal features that only mediate unconsciousness. The NCC thus signify a special neuronal role for consciousness and entail a neuronal dichotomy of consciousness vs. unconsciousness. Specialness and dichotomy are thus now “located” within the brain itself, that is, in terms of two sets of neuronal features and mechanisms.

Without going into detail, these specific neuronal mechanism include, to name just a few of the various suggested ones, information integration (Tononi et al., 2016), recurrent processing (Lamme, 2018), access to global workspace (Dehaene et al., 2017), embodiment (Tallon-Baudry et al., 2018), higher-order cognition (Lau and Rosenthal, 2011), predictive error minimization (Hohwy, 2013), temporo-spatial dynamics (Northoff and Huang, 2017; Northoff, 2019), or subcortical mechanisms and affect/emotion as emphasized by Panksepp (1998a,b), Damasio (2018) and Solms (2018, 2019).

How can we take into view the specialness of the neuronal mechanisms of mental features and their dichotomy to those of non-mental features? That is possible only by taking a view from within the brain itself and, more specifically, from within the neuronal mechanisms supposedly underlying mental features. One thus presupposes a “vantage point from within brain” in neuroscience. The mento-centric view of psychology is thus replaced by a neuro-centric view of mental features in neuroscience. However, despite the difference between mento- and neuro-centric views of mental features, both psychology/cognitive science and neuroscience still presuppose a rather egocentric vantage point, e.g., that is, from within either mind or brain which entails what, further down, I describe as brain-world relation. This marks both approaches to mental features as pre-Copernican (see Figure 2B).

Some approaches may want to argue that they are not neuro-centric as they, instead of the brain, presuppose information (Tononi et al., 2016), cognition (Lau and Rosenthal, 2011), or the body (Noe, 2004; Thompson, 2007; Blanke et al., 2015; Tallon-Baudry et al., 2018) as primary basis of mental features like consciousness. True indeed, these approaches are no longer neuro-centric. That does not relieve them of their ego-centric character though. The ego-centric approach is now transferred from the brain to body, cognition, or information—they are thus body-centric, information-centric, and cognition-centric and therefore be characterized by “vantage points from within information, cognition or body.” As in the neuro-centric approaches, both specialness of mental features and their dichotomy to non-mental features are still preserved in these approaches (which shall not be elaborated in detail here) this marks them as pre-Copernican.

### Vantage Point From Beyond Brain I—Brain-World Relation vs. World-Brain Relation

One may now want to raise the question for a post-Copernican approach to the brain, a vantage point from beyond brain, and how that will look like. Like earth and human species, the brain and mental features would then no longer be conceived as special nor as dichotomous to the world. I here briefly want to formulate the criteria of such vantage point from beyond brain which then will be explicated in a more concrete way in the subsequent parts.

A vantage point from beyond brain must allow us taking into view that what lies beyond the boundaries of the brain itself and, even more important, how that shapes the brain. More specifically, we need to consider how the world and its external dynamic shape and impact the brain as featured by its own internal dynamic (see below for the exact meaning of dynamic). Accordingly, we need to take into the relationship between world and brain, that is, how the world shapes the brain—this is what I recently described as “world-brain relation” (Northoff, 2016a,b,c,d, 2018; see Figure 2C).

The world-brain relation needs to be distinguished from the reverse relationship, that is, how the brain shapes and cognizes the world—this is described as “brain-world relation” (Northoff, 2016a,b,c,d, 2018; see also Figure 2B). The distinction between world-brain relation and brain-world relation is important in both aspects, empirically and methodologically.

Empirically, the brain-world relation entails that the brain imposes itself upon and shapes the world—this is the case in especially cognition and action. This is different in the case of the world-brain relation where the world primarily shapes the brain rather than the latter shaping and imposing itself upon the former. That is empirically supported by data showing how, for instance, early life events in the world shape the brain’s temporo-spatial dynamic, e.g., its degree of entropy in ventromedial prefrontal cortex (Duncan et al., 2015), and internally-guided decision making (e.g., N200 in EEG; Nakao et al., 2013) later in adulthood.

Yet another empirical example of how the world shapes the brain is the phenomenon of entrainment where the neural activity of the brain actively adapts to the events in the environment like the rhythm of music or tone sequences (Lakatos et al., 2013; van Atteveldt et al., 2015). We all know such alignment of our brain to the world only too well as when we, for instance, unconsciously, tap our feet in the rhythm of the background music.

Together, these examples show that the brain’s neural activity is strongly shaped by the world by either the latter imposing itself upon the former, e.g., as in the life events, or, alternatively, by the brain actively adapting to the world, e.g., as in entrainment. Common to both examples is that the world’s external dynamics shapes the brain’s internal dynamics—we, therefore, speak of “world-brain relation” as distinguished from brain-world relation where the brain’s internal dynamics shapes and imposes itself upon the world’s external dynamics. Note that the distinction of world-brain relation vs. brain-world relation is not an absolute and mutually exclusive. Instead, world-brain relation and brain-world relation stand in a dynamic balance with each other—their conceptual distinction is thus relative (rather than absolute).

Finally, one may be surprised why we almost completely neglect the body here. Recent data show that the brain and its internal dynamic align to the body’s dynamics in more or less the same way as it aligns to the world’s external dynamics. For instance, various studies by the group around Tallon-Baudry et al. (2018) demonstrated that the brain’s internal dynamics aligns its phase onsets to the onsets of the heartbeat—one can thus speak of “body-brain relation” (Northoff, 2018). The brain thus recruits the same mechanisms for its alignment, e.g., relation to the body as it employs when synchronizing with its external environment, e.g., the world. Therefore, we assume that the body-brain relation can be subsumed (conceptually) under the more extended world-brain relation (given also that the body is part of the world; Northoff, 2018 for details).

### Vantage Point From Beyond Brain II—Post-Copernican Vantage Point in Neuroscience

The distinction of world-brain relation and brain-world relation carries major methodological implications. Featuring how the brain shapes and imposes itself upon the world, the brain-world relation conceives the brain as center and the world as periphery. This presupposes a vantage point from within brain. One consequently comes to the assumption of the specialness of the brain (as distinguished from non-brains) and its dichotomy to the world—this resembles the pre-Copernican vantage points in astronomy and biology. Since mental features are supposed to be caused by or identical with the brain (see Solms, 2019 for the difference between causal theories and dual-aspect monistic accounts of mental features), the specialness of the brain and its dichotomy to the world do then also apply to mental features like consciousness, self, and affect (and other mental features).

This is different in the case of the world-brain relation. Unlike the brain-world relation, the world-brain relation is based on that what happens beyond the boundaries of the brain, e.g., the world’s external dynamics, and how it shapes the brain’s internal dynamics. That can be taking into view only when presupposing a post-Copernican vantage point from beyond brain—the brain is then no longer special (when compared to non-brains) nor dichotomous to the world (see Figure 2D).

Presupposing such vantage point from beyond brain, we can then take into view how mental features extend beyond the brain as they may be traced to and based on the world and how it shapes the brain, e.g., world-brain relation. Consciousness, self, affect and other mental features may consequently no longer be conceived as exclusively neuronal but neuro-ecological (see below for details). Most important, this implies that mental features are no longer special nor in dichotomous relation to the world. Presupposing a vantage point from beyond brain, we will now, in the next part, sketch (albeit very preliminary) such post-Copernican view of both brain, e.g., in terms of world-brain relation and free energy (third part), and mental features (fourth part; for more details, see Northoff, 2016a,b,c,d, 2018).

We here pursue a two-step procedure to explicate such post-Copernican approach. First, we explicate and detail what is meant by world-brain relation in more biological detail by characterizing it by the free-energy principle of Friston (2010). This amounts to a post-Copernican view of the brain (third part). Second, being characterized by free energy, we are then able to link world-brain relation in a necessary way to mental features; such necessary connection is provided by dynamic and more specifically temporo-spatial features as “common currency” of world, brain, and mental features (Northoff, 2018, 2019). This entails a post-Copernican view of mental features.

## Part III: Post-Copernican View of the Brain—Free Energy

### Free Energy I—Neuro-Ecological and Biological View

The organism and its brain are not isolated from the world but deeply embedded within and dependent upon the world, e.g., its respective environmental context (which is the meaning in which understand the concept of “world” in the following). There is interaction between the world’s external dynamics and the brain’s internal dynamics. Both can interact in a bilateral or mutual way in that the brain’s internal dynamics can conform to the world’s external dynamics (“perception” as Friston says), e.g., world-brain relation, or, conversely, the world’s external dynamics can conform to the brain’s internal dynamics (“action” as Friston says), e.g., brain-world relation (Friston and Stephan, 2007; Friston, 2010; Bruineberg and Rietveld, 2014; Bruineberg et al., 2018a,b).

How is such bilateral interaction between world/environmental context and brain mediated? That is the moment where Friston’s free energy principle comes in. Roughly, free energy provides the commonly shared reference of both world/environmental context and brain according to which they adjust and relate to each other. The interaction of organism/brain and world/environmental context is characterized by the attempt to minimize the amount of free energy that is discrepant between both systems (Friston and Stephan, 2007; Bruineberg and Rietveld, 2014). Friston thus speaks of “free energy minimization” as basic principle of the organism’s life that specifically characterizes the brain (Friston and Stephan, 2007).

We need to be careful though. The concept of “free energy minimization” can be understood in different ways. One most commonly held assumption is that free energy minimization is a guiding principle within the brain itself; the different layers of neuronal activity and its hierarchy do then aim to minimize their amount of free energy against each other. Here, free energy minimization is taken to be closely linked to (if not almost identical with) predictive coding as central computational mechanism of the brain’s neuronal activity (Hohwy, 2013; for an excellent discussion, see Bruineberg et al., 2018a). This amounts to a neuronal view of free energy which, conceptually, merges well with what I described as brain-world relation.

Such neuronal view of free energy stands in contrast to the here sketched more biological view. The biological view conceives free energy as basic principle and common reference for the interaction of world/environmental context and organism/brain. Free energy here is no longer restricted to the brain itself and confined within its boundaries. In contrast, free energy is supposed to operate at and beyond the boundaries of the brain by guiding its interaction with and attunement to the world/environmental context. That allows to view free energy as the central principle and reference for regulating the homeostasis of the organism/brain with the world/environmental context. I here follow such biological and neuro-ecological view of free energy (see Bruineberg and Rietveld, 2014; Bruineberg et al., 2018a,b as it aligns well with what I describe as world-brain relation (see above).

### Free Energy II—Generative Model and Variational Density

How can the organism and its brain access and modulate free energy relative to their respective environmental context? Friston assumes that the organism/brain’s internal dynamics can be characterized by two features, that is, generative model and variational density. In a nutshell, the generative model describes the probability of co-occurrence between the brain’s internal states and the environmental context’s external state. Importantly, the generative model does not amount to a neuronal representation of the world within the brain itself. Instead, the generative model refers to the long-term stochastic regularities in the relationship between world/environmental context and organism/brain (see Bruineberg et al., 2018a who emphasize this point).

Since the generative model does not provide a neuronal representation of the world, it can not be thought of as a model of the world that the organism and its brain create within themselves. Instead, the organism and its brain are by themselves a model of the world and, more specifically, “being a model of their econiche” (Bruineberg and Rietveld, 2014). The concept of generative model describes the organism’s eco-niche within the world by free energy—this entails a neuro-ecological rather than purely neuronal view of generative model. Based on such neuro-ecological understanding of generative model, free energy can be thought as basic biological principle that provides the coupling or attunement between the organism/brain’s internal dynamics and the world/environments’ external dynamics.

How can the organism and its brain actively modulate their free energy as to conform to their respective environmental context by minimizing free energy? Friston takes variational density as proxy for probability distribution within the organism itself including its body (like temperature) and brain [e.g., its “perception/action,” as Friston says, which is interpreted in terms of “readiness states” by Bruineberg and Rietveld, 2014 as it does not really imply actual (or real) but only possible (not yet realized) perception/action]. Variational density is encoded in the organism/brain’s internal dynamics (see below for details of this point) whose probability distribution can be changed to minimize free energy in its relationship to the world/environmental context.

Variational density, reflecting the brain’s internal dynamics, is, for instance, changed by anticipation. If the organism can, through its brain, anticipate the state of the world/environmental context, the free energy between world/environmental context and organism/brain is minimized and thus low. In that case, there is strong coupling and high attunement of world and brain—that is, for instance, the case when dancing to the rhythm of the music. If, in contrast, anticipation remains impossible, free energy is rather high. That is manifest in low coupling with less attunement of world and brain—in that case, one cannot get into the rhythm of the music.

### Free Energy III—Vantage Point From Beyond Brain

Free energy featured by generative model and variational density can be understood as the basic biological principle that guides the relationship of world/environmental context and organism/brain, e.g., world-brain relation as I coined it above. Specifically, free energy can be understood as the biological mechanism that establishes relationship between world and brain. Taken in such a way, free energy must be understood as intrinsically neuro-ecological and biological rather than as purely neuronal and neuroscientific.

Such neuro-ecological view of the brain as part of the world with their relation established by free energy is only possible by presupposing a vantage point that allows to take into view that what happens beyond the boundaries of the brain. The here suggested neuro-ecological and biological view of free energy (see Bruineberg et al., 2018a,b) thus presupposes a post-Copernican vantage point from beyond brain. Like in the cases of earth and human species in astronomy and biology (see above), such post-Copernican vantage point from beyond brain radically changes our view of the brain in neuroscience.

The brain as an organ that aims to minimize free energy is no longer special when compared to other organs, i.e., non-brains, which, being biological adhere to the same principle. Nor does the brain stand in a dichotomous relationship to the world anymore as it is intimately coupled to the latter through free energy minimization. This specifies and presupposes what I described as world-brain relation. If, in contrast, one shifts towards a neuronal and neuroscientific concept of free energy, one’s view remains restricted to the brain itself without taking into view that what happens beyond the brain, that is, how it is related and coupled to the world. This entails a pre-Copernican vantage point from within brain where the brain remains special and stands in a dichotomous relation to the world thus presupposing brain-world relation (rather than world-brain relation).

## Part IV: Post-Copernican View of Mental Features—Temporo-Spatial Dynamic as “Missing Ingredient” and “Common Currency”

### Mental Features I—Biological and Neuro-Ecological

**Figure 3:**
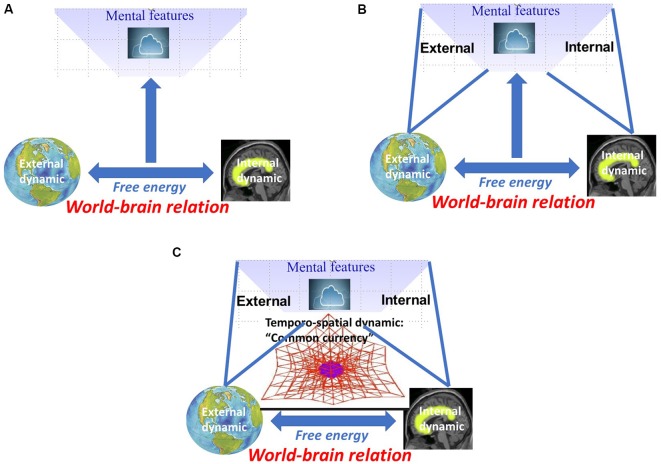
**(A)** Neuro-biological characterization of mental features by free energy between world and brain. **(B)** Neuro-ecological characterization of mental features by internal-external relation. **(C)** Temporo-spatial dynamic as “common currency” of world-brain relation, free energy, and mental features.

I characterized the world-brain relation by free energy that allows for relating and coupling world and brain. This raises the question of how such world-brain relation, as based on free energy, stands in relation to mental features like consciousness, self, and affect. Applied in this sense, free energy provides a biological and neuro-ecological characterization of mental features (see Figure 3A).

Such biological characterization in terms of free energy has indeed been suggested by various authors for different mental features. Without going into details of the various approaches, I here just mention some (which neglects various others). One model of consciousness that takes the computational mechanisms of free energy as a starting point is the “projective consciousness model” (Rudrauf et al., 2017). Yet others have applied the free energy principles to dreams (Hobson et al., 2014). Moreover, free energy has even been assumed to provide an answer to the hard problem of consciousness (Solms, 2019), that is, why is there consciousness rather than non-consciousness (Chalmers, 1996).

Yet another mental feature where free energy has been intensively applied is the self. Bodily approaches to the self, e.g., somatic self, extensively rely on free energy (Seth and Tsakiris, 2018). More generally, the self as such, e.g., as distinguished from non-self, has been associated with free energy by Friston himself (Friston, 2018). Yet other recent approaches to the self like the dynamic pattern theory of self (Gallagher and Daly, 2018) and the subjective self (“I” vs. “me”) strongly rely on free energy.

Finally, affect and emotion have also been related to free energy. One major proponent of such approach is Solms (2017, 2018, 2019). He associates the subcortical regions of the brain, as relying on Panksepp (1998a,b) and Damasio (2018), with affect and especially its subjective first-experiential features as paradigmatic and most basic manifestation of consciousness. Affective and its subjective feature are, in turn, assumed to be closely linked to free energy minimization with the environment (Solms, 2017, 2018, 2019). Hence, Solms links free energy to affect and consciousness in a unique way. Yet another proponent is Seth who develops an embodied theory of the free energy-based concept of active inference and how that relates to emotions (Seth and Friston, 2016).

Taken together, free energy is conceived central for mental features including consciousness, self, and affect (and others not mentioned here). If free energy characterizing the relation between world/environmental context and organism/brain is indeed central for mental features, one would expect the latter to be neither purely external, e.g., ecological, nor purely internal, e.g., neuronal, either. Instead, one would then assume mental features to be intrinsically neuro-ecological rather than merely neuronal.

### Mental Features II—Internal-External Relation

Free energy regulating the relation of world/environmental context and organism/brain relates the former’s external dynamics and the latter’s external dynamics. If mental features do indeed depend upon free energy, one would expect them to signify different forms of internal-external relations (thus reflecting their neuro-ecological rather than neuronal characterization). This raises the question of how we can characterize the relationship between internal and external dynamics on both biological and mental levels.

On the biological level, the internal dynamics of the organism/brain is coupled to the external dynamics of the world/environmental context. Both share mutual information which is manifested in what Friston (2010) describes as “generalized synchrony” (as distinguished from representation). Generalized synchrony refers to the “coupled dynamics” between two systems who synchronize their different time scales with each other like the Huygens clocks where two clocks synchronize their time scales with each other over time—such synchronization can then be conceived as manifestation of free energy minimization (Bruineberg et al., 2018a).

The same kind of synchronization now happens, analogously, in the relationship between world/environmental context and organism/brain when they couple with each other. For instance, when we tap our foot in the rhythm of the music, our brain and its internal dynamic entrain to the external dynamic of the latter. Neuronal investigation show, for instance, the brain synchronizes its phase onsets with those of continuous external stimuli (Lakatos et al., 2013) which seems to be disrupted in schizophrenia (Lakatos et al., 2013). One can thus see how, on the biological level, free energy allows for establishing synchronization between external and internal dynamics of world/environmental context and organism/brain.

We now assume that such synchronization between internal and external dynamics is also central for establishing relation between internal and external contents in mental features, i.e., internal-external relation. For instance, Honey et al. (2017) recently showed how perception, memory, and others can be characterized by different forms of internal-external relations. Yet another example is consciousness. Inner time consciousness, for instance, can be characterized by the relation between the subject’s own “inner time speed” and how it perceives “outer time speed” (Fuchs, 2013). Usually, inner and outer time speed are somewhat in synchrony in our consciousness with both mutually adjusting and coupling to each other (Fuchs, 2013; see Figure 3B).

However, they can also differ and thus be non-adjusted. That is, for instance, the case in psychiatric conditions like depression and mania. In the case of depression, inner time speed in consciousness is too slow while the subjects perceive outer time speed, e.g., the time speed in the world, as too fast (Northoff, 2018). While the reverse happens in mania where subjects’ inner time speed is fast (as manifested in fast action and psychomotor agitation) while they perceive outer time speed, relative to their abnormally fast inner time, as too slow—they thus become impatient (Northoff, 2018). Accordingly, as exemplified by our example of inner and outer time speed, consciousness can be characterized by specific relation of internal and external dynamic in our subjective experience, i.e., internal-external relation.

The same holds for other mental features like self and affect. The self is based on relating external stimuli and objects in the environment to the internal dynamics—this has been described as self-related processing (Northoff et al., 2007; Northoff, 2011, 2016a,b,c,d). For instance, even our own name is nothing but a collection of syllables which need to be put together by our brain to shape what we call our own name—that is possible only by processing the syllables more strongly related to the own brain and its internal dynamics than those of another person’s name. Accordingly, what we describe as self-related (like our own name) reflects a certain constellation between the brain’s internal dynamics, e.g., its spontaneous activity and the environment’s external dynamics, e.g., the syllables. If that specific internal-external relation is disrupted, as in schizophrenia, we may lose our sense of self (Northoff and Duncan, 2016). The self can thus be featured as relational and neuro-ecological (Northoff, 2016a,b,c,d).

Yet another example of internal-external relation shaping the self is transcultural differences. It has been well established that the self is constructed in a more inter-dependent, e.g., social way, in far eastern cultures (Markus and Kitayama, 1991; Han and Northoff, 2008). In contrast, the self is constructed in a more independent, e.g., isolated way in western culture. Most interestingly, such difference between inter- and in-dependent self is accompanied by neuronal differences (Han and Northoff, 2008; Han et al., 2013). Hence, the self in different cultures is neither purely internal nor exclusively external but is constituted by different degrees or balances of internal-external relation.

### Temporo-Spatial Dynamic I—World-Brain Relation and Free Energy

One may now raise the question of why and how the coupling of world and brain, the world-brain relation, can give rise to mental features. This question focuses especially on the subjective experience and its phenomenal features like qualia, intentionality, transparency, unity, et cetera (for details Northoff, 2014a,b), that characterize all mental features like consciousness, self, and affect. In order for the world-brain relation and its free energy to yield mental features, both must share something that first and foremost makes possible the transformation of the former into the latter. This is the search for what we recently described as “common currency” (Northoff, 2019).

A “common currency” allows for exchange and mutual adaptation. Consider for instance the US dollar that provides the “common currency” between the different currencies in the global economy. By referring their own currency to the US-dollar, different countries can exchange and trade goods. Goods can thus be transformed from one country to another country. We now assume that such “common currency” is also central in transforming the neuro-ecological activity of world-brain relation, e.g., free energy, into mental features like consciousness, self, and affect (for details, see Northoff, 2019).

What is the “common currency” of world-brain relation and its characterization by free energy on the one hand mental features on the other? To address this question, we first have to briefly address the notion of time and space and, secondly, how they characterize both world-brain relation and free energy. Note that the conceptions of time and space are here not understood in the way we perceive or cognize time and space in terms of discrete points in time and space (see for instance, Buzsáki and Llinás, 2017; Drayton and Furman, 2018). Instead, we rather refer to time and space in a dynamic sense as in dynamical system theories where time and space are rather described in terms of attractors and trajectories (Cocchi et al., 2017) this amount to what we refer to as “temporo-spatial dynamic” (see also Northoff and Huang, 2017; Northoff, 2019).

Let us be more concrete. The brain constructs such dynamics in terms of different frequencies with oscillations and fluctuations (Buiszaki, 2006) that show a specific temporal structure with long range temporal correlations (LRTC) and scale-free activity (Linkenkaer-Hansen et al., 2001; He et al., 2010). As they connect different points in time by operating across different temporal scales, LRTC can be conceived as an example of temporal relation. Specifically, LRTCs and scale-free activity reflect the relationship between different frequencies and thus model different points in time relative to each other (see Northoff, 2019 as well as Northoff and Huang, 2017 for more details on the brain’s temporo-spatial dynamic).

Importantly, the brain’s construction is closely aligned to the way how it couples and relates to its respective environmental context, that is, in terms of free energy. This is reflected in the fact that the assumption of space and time takes also center stage in formulations in the free energy principle. That follows because the variational free energy is defined in terms of a generative model (see above) and the generative model includes information about the relation of world and brain, e.g., their degree of stochastic matching or convergence (Friston et al., 2006). This leads to the notion of *deep temporal models* that possess a necessary *temporal thickness* or depth (Seth and Friston, 2016). Together, albeit only hinted upon, both free energy and brain can be characterized by an elaborate temporo-spatial dynamic.

### Temporo-Spatial Dynamics II “Missing Ingredient” and “Common Currency” of Neuronal and Mental Features

Mental features can also be characterized by time and space, that is, what phenomenological accounts describe as “spatiotemporality.” Consciousness, for instance, can be characterized by a “stream” and the inclusion of presentation, prospection, and retrospection (James, 1890a,b; Husserl, 1921) this has been subsumed under the umbrella of “inner time consciousness” (Husserl, 1921; Fuchs, 2013; Northoff, 2014a,b). The same holds analogously for space (Ferri et al., 2015); for that reason, philosophers characterize consciousness by “spatiotemporality” (James, 1890a,b; Husserl, 1921; Zahavi, 2005; Fuchs, 2013).

Recent modeling further supports such view by showing that the “spatiotemporality” of mental features can be understood in dynamical terms, that is by temporo-spatial dynamics in terms of virtual trajectories in what has been described as “phenomenal space” (Prentner, 2019). Such dynamic temporo-spatial view of mental features like consciousness and self (for the latter, see Wolff et al., 2019) thus replaces the non-temporal view of mental features in traditional philosophy and the more recent static temporal approach to mental features in terms of perception and cognition.

We are now ready to address the quest for the “common currency.” Albeit tentatively and laid out in more detail elsewhere (Northoff, 2014a,b, 2018; Northoff and Huang, 2017; Northoff, 2019), we assume that temporo-spatial dynamical features provide the link between neuro-ecological and mental levels—the former’s temporo-spatial dynamic is thus supposed to be manifest in the latter’s “spatiotemporality.” We, therefore, suppose that temporo-spatial dynamic may be a good candidate to provide the “missing ingredient” (Lamme, 2018) and “common currency” (Northoff, 2019) of world-brain relation, free energy, and mental features (see Figure 3C).

How can we lend more concrete empirical support to the assumption of temporo-spatial dynamics providing the “common currency” of neuronal and mental features? This has recently been put into more specific terms when, for instance, assuming that the scale-free activity of the brain’s spontaneous activity transforms into more or less analogous scale-freeness with the integration of different time scales on the psychological level of consciousness, e.g., its arousal or level/state (Tagliazucchi et al., 2013, 2016; Northoff, 2017; Cavanna et al., 2018). Analogously, recent studies demonstrated that the self is also mediated by temporo-spatial features of the spontaneous activity like scale-free activity, autocorrelation window and cross-frequency coupling which may be in analogous way manifest on the psychological level (Huang et al., 2016; Wolff et al., 2019). The different affects as described by Panksepp (1998a,b) and Solms (2017, 2018, 2019) may then also be described by different forms of spatial and temporal coordinates in their subjective experience.

Yet another instance where temporo-spatial features transform from the neuronal to the mental level are psychiatric disorders like autism (Damiani et al., 2019) and bipolar disorder (Martino et al., 2016, 2018) where recently “Spatiotemporal Psychopathology” (Northoff, 2016a,b,c,d, 2017, 2018; Fingelkurts and Fingelkurts, 2019) has been proposed. This is supported by data on consciousness, self, and bipolar disorder (for details, see Northoff, 2019).

For instance, the above mentioned inner and outer time speed changes in the consciousness of depressed and manic patients are related to corresponding time speed changes in the neuronal activity in those networks mediating inner and outer time experience/perception (Northoff, 2018). Together, these examples support the view that temporo-spatial dynamics provides the “common currency” of neuronal and mental features in both healthy and pathological states.

### Temporo-Spatial Dynamics III—Post-Copernican Approach to Mental Features

Why do we require a novel methodological strategy for postulating temporo-spatial dynamics as “common currency” of neuronal and mental features? I assume that this is only possible by presupposing a post-Copernican approach with a vantage point from beyond brain. Let me sketch that briefly.

The concept of “common currency” provides the necessary (rather than contingent) connection of neuronal and mental features that so far remained elusive to us. We simply do not know how neuronal and mental features are intrinsically linked to each other—we miss something, the “missing ingredient” (Lamme, 2018). I suggest in this article that the lack of insight into the necessary connection and thus the “missing ingredient” is due, at least in part, to our pre-Copernican methodological strategy.

Specifically, our currently pre-Copernican ego-centric vantage point from within brain (or within body, information, or cognition; see above) prevents us from taking into view that what happens beyond the brain in world (and body) and how that shapes the brain’s neuronal activity in such that it is intrinsically and thus necessarily connected to mental features. We consequently assume mental features to be special as related to specific neuronal mechanisms (like the NCC) as distinguished from those underlying others, e.g., non-mental features. This renders impossible to take into view the necessary connection of neuronal and mental features. Moreover, that puts mental features in a dichotomous relation to the world and its non-mental features.

That changes once one shifts the pre-Copernican vantage point from within brain to a post-Copernican vantage point from beyond brain. The vantage point from beyond brain allows taking into view that what happens beyond the boundaries of the brain in the world, e.g., world-brain relation as featured by free energy, and how that, e.g., its temporo-spatial dynamics, shapes and yields mental features with their own spatiotemporality. Being a viable candidate to provide the “common currency,” temporo-spatial dynamics establishes intrinsic and thus necessary connection of neuronal and mental features.

Most importantly, mental features are then no longer conceived as special (when compared to non-mental features) but rather non-special. Moreover, being temporo-spatial, mental features no longer stand in dichotomous relationship to the world but in a “temporo-spatial continuum” this specifies and explicates what, in more general terms, has been described as “embeddedness/enactivism” or “deep continuity of mind and life” (Thompson, 2007; Clark, 2013). Most important, such view of mental features resembles very much our current post-Copernican views of both earth and human species including their continuous relationship to universe and evolution that were established by Copernicus and Darwin.

## Conclusion

I here propose a novel methodological strategy on how to approach brain and mental features. Relying on Copernicus and Darwin, I advocate changing our currently rather pre-Copernican vantage from within brain to a post-Copernican vantage point from beyond brain. This allows us taking into view that what happens beyond the boundaries of our brain, e.g., in world and body as described in the concept of world-brain relation, and how that shapes the brain in such way that it can yield mental features. Relying on Friston, I characterize such world-brain relation in a biological way by the free-energy principle. That renders the brain as intrinsically neuro-ecological rather than merely neuronal.

Such post-Copernican vantage point from beyond brain allows us taking into view that both free energy of world-brain relation and the brain itself are characterized by temporo-spatial dynamics. Based on empirical evidence, such temporo-spatial dynamic is also manifest in mental features, e.g., their spatiotemporality. I therefore conclude that a post-Copernican approach to the brain, e.g., a vantage point from beyond brain, allows us taking into view temporo-spatial dynamics as a viable candidate of the so far elusive “missing ingredient” and “common currency” of neuronal (or better neuro-ecological) and mental features (Northoff, 2019).

I conclude that neuroscience may benefit from a shift in its vantage point from within brain to beyond brain in its methodological approach to mental features. That amounts to nothing less than a Copernican turn or revolution in neuroscience akin to the ones in both astronomy and biology. Like in the latter disciplines, such methodological shift can, as I propose, provide a novel framework for neuroscience that will turn its search for the neural basis of mental features into a true and major success story at the beginning of the 21st century.

## Author Contributions

GN being the sole author, contributed all parts of the article himself.

## Conflict of Interest Statement

The author declares that the research was conducted in the absence of any commercial or financial relationships that could be construed as a potential conflict of interest.
